# Extracellular vesicles of Gram-negative and Gram-positive probiotics

**DOI:** 10.71150/jm.2506005

**Published:** 2025-07-31

**Authors:** Yangyunqi Wang, Chongxu Duan, Xiaomin Yu

**Affiliations:** 1 Queen Mary School, Jiangxi Medical College, Nanchang University, Nanchang 330006, P. R. China; 2 School of Basic Medical Science, Jiangxi Medical College, Nanchang University, Nanchang 330006, P. R. China; 3 Medical Experimental Teaching Center, School of Basic Medical Science, Jiangxi Medical College, Nanchang University, Nanchang 330006, P. R. China

**Keywords:** probiotics, extracellular vesicles, immunomodulation, biofunction, host-interaction, therapeutic potential

## Abstract

Extracellular vesicles derived from probiotics have received considerable attention for their pivotal role in bacterial‒host communication. These nanosized, bilayer-encapsulated vesicles carry diverse bioactive molecules, such as proteins, lipids, nucleic acids, and metabolites. Currently, ample evidence has emerged that probiotic extracellular vesicles may modulate several processes of host physiological hemostasis and offer therapeutic benefits. This review examines the biogenesis, composition, and immunomodulatory functions of probiotic-derived extracellular vesicles in probiotic–host interactions, highlighting the therapeutic potential of probiotic extracellular vesicles in the diagnosis and treatment of conditions such as cancer and inflammatory bowel disease. We further summarize the techniques for the separation and purification of extracellular vesicles, providing a methodological foundation for future research and applications. Although the field of probiotic extracellular vesicle research is still in its infancy, the prospects for their application in the biomedical field are broad, potentially emerging as a novel therapeutic approach.

## Introduction

Probiotics, which are live microorganisms that are beneficial to the host, have been widely studied for their ability to affect gut health and their potential in treating various diseases. Although their benefits are well known, the mechanisms behind their extensive effects, especially the role of extracellular vesicles (EVs), are not fully understood and are promising areas for further research.

EVs, such as membrane vesicles (MVs) and outer membrane vesicles (OMVs), are nanoscale structures, usually 20–400 nm in size, that are released by bacteria. They play a vital role in microbial–host interactions. Gram-negative (G-) bacteria, whose cell walls contain a substructure called the outer membrane, release OMVs. In contrast, Gram-positive (G+) bacteria, which lack an outer membrane, produce MVs directly from the cytoplasmic membrane (Vicente-Gil et al., 2024). EVs can be released naturally, budding from the outer membrane without disrupting its integrity, or during cell death or lysis. Given their nanoscale size, EVs can cross cellular barriers and interact with the immune system, making them important for bacterial survival, communication, and potential applications in vaccines and antibacterial therapies.

Both pathogenic microorganisms and probiotics can spontaneously produce EVs, and it is generally believed that probiotic-derived EVs have beneficial effects, whereas pathogen-derived EVs negatively impact the host. Recent studies have highlighted the potential of probiotic EVs in treating and diagnosing conditions such as pancreatic and cervical cancer and colonic lesions (Karamitopoulou et al., 2023). Despite a surge in probiotic usage over the past two decades, research on the therapeutic and preventive roles of probiotic EVs remains limited. While much focus has been placed on EVs from G- bacteria, most probiotics are G+, indicating a need for further investigation. The study of EVs is pivotal for revealing the intricacies of disease-related microorganisms, including the etiology instigated by bacteria, the mechanisms of probiotic action, and the interplay between hosts and pathogens.

This review comprehensively summarizes the latest research on probiotic EVs. It covers biosynthesis, composition, immunomodulatory functions, therapeutic potential, and host interactions. The article also highlights challenges in EVs heterogeneity, disease-specific mechanisms, and safety assessment. EVs research is crucial. It not only holds promise for disease treatment and prevention but also serves as a tool to understand probiotic–host molecular interactions. Future research must address these issues for clinical translation. A deeper understanding of EVs functions and mechanisms will enable the development of more precise diagnostic and treatment methods, promoting personalized medicine and precision therapy.

## Biological Effects of Probiotic EVs

EVs are nanoscale structures crucial for biological functions. Their unique edge crosses the intestinal barrier, enters the bloodstream, and reaches other body parts. The high biocompatibility and low immunogenicity of probiotics to the host make the ability of EVs to home to the target tissues a key material basis in probiotic research. Studies have shown that probiotic EVs are vital for immunomodulation and disease protection, indicating their potential to impact host defense and disease outcomes. This knowledge could increase the therapeutic efficacy of probiotics, furthering their important role in human health and disease management. Building upon the regulation of the immune system by probiotic EVs through macrophages, B-cell activation, innate T-cell responses, traditional T-helper responses, and T-regulatory mechanisms, research on other probiotic strains has advanced and will be discussed below.

## Immunomodulatory Effects of Probiotic-derived EVs on Macrophages

Probiotic-derived EVs can induce macrophages to polarize toward the M2 phenotype. In pathological states, macrophages are mainly in the M1 phenotype, which is pro-inflammatory with bactericidal and pathological clearance functions and is activated by interferon-γ (IFN-γ) and lipopolysaccharide (LPS) via Jak/STAT1 pathway. Probiotic-derived EVs can dynamically polarize M1 to M2, reducing M1 polarization and increasing M2 polarization for anti-inflammatory and immunoregulatory effects related to tissue repair; these effects are activated by IL-4, IL-13, and IL-10 and promote M1/M2 polarization through the STAT6, STAT3, and multiple pathways (Wang et al., 2014; Yunna et al., 2020). *Lactobacillus plantarum* strain LP25, a member of the lactic acid probiotic family, MVs produced by LP25 (Lp25_MVs) modulate immune system by promoting M2-type macrophage polarization and upregulating IL-10 and arginase-1 (Fan et al., 2024). *Lactobacillus murinus* derived MVs mitigate deoxynivalenol-induced inflammation and rebuild intestinal homeostasis by increasing the secretion of IL-10, thereby activating macrophage toll-like receptor 2 (TLR-2) and M1-to-M2 polarization. Coequal effects were also identified in MVs derived from *Limosilactobacillus mucosae* (Liu et al., 2020, 2023; Vergadi et al., 2017).

**Fig. 1.**
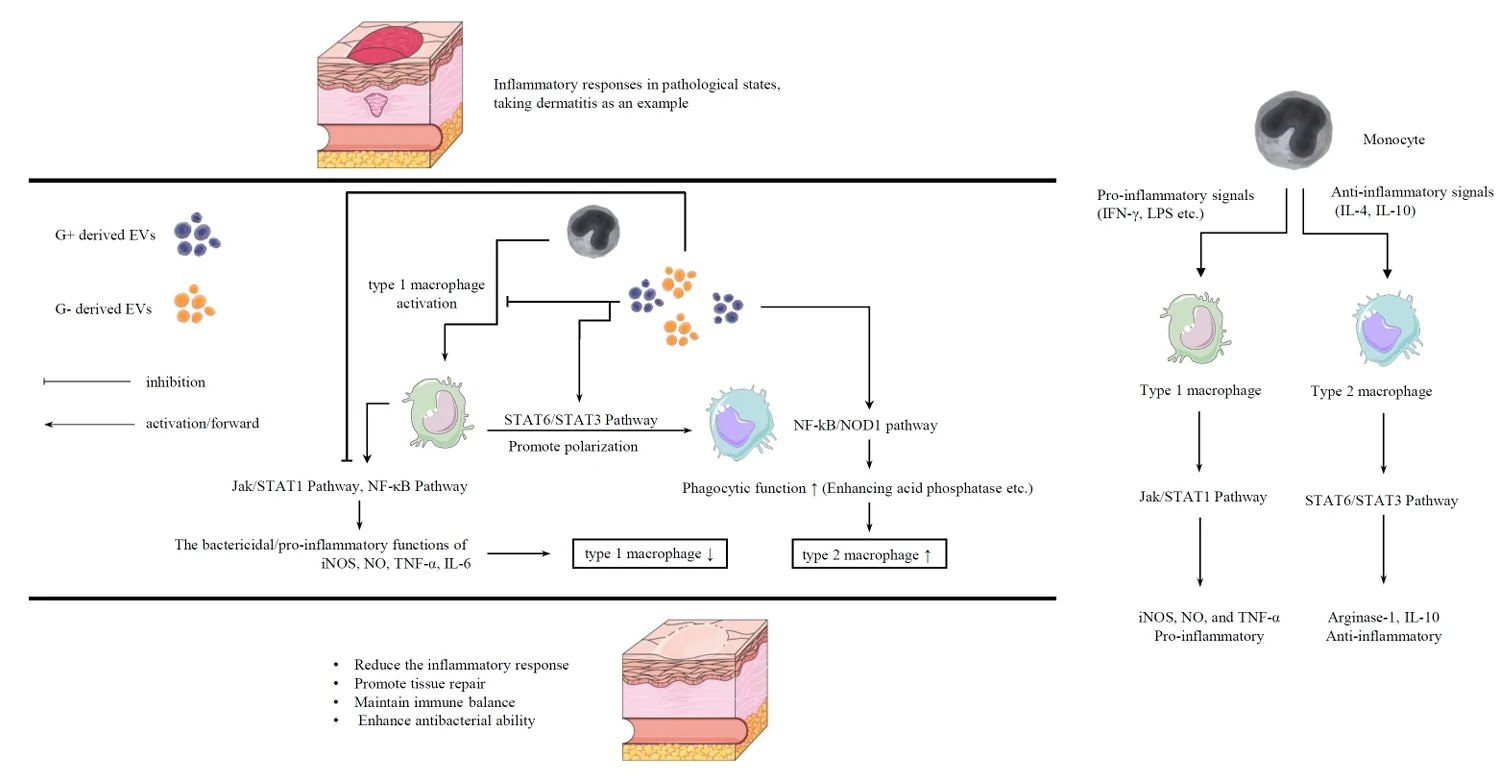
Intervention of probiotic-derived extracellular vesicles in innate immune regulation based on macrophages. The enhancement of innate immunity by probiotic outer membrane vesicles was mainly due to its effect on macrophages. Probiotic outer membrane vesicles promote the polarization transformation of M1-type macrophages to M2-type macrophages, and inhibit the function and proliferation of M1-type macrophages. M2 macrophages can enhance immune function by activating MAPK, NF-κB cell pathway and up-regulating MHC expression, and release anti-inflammatory substances such as IL-4, IL-10, Arg-1, etc., to achieve the effect of anti-inflammation.

*Escherichia coli* Nissle 1917 (EcN), a key G- probiotic and model organism in OMVs research, was the first source of *E. coli*-derived OMVs discovered in humans. Studies have shown that OMVs produced by EcN (EcN_OMVs) increase the immune-related enzymatic and phagocytic activities of RAW 264.7 macrophages. The activities of acid phosphatase, nitric oxide and nitric oxide synthase significantly increase when RAW 264.7 macrophages are stimulated with low to moderate levels of EcN_OMVs, indicating macrophage activation (Hu et al., 2020). Zhu et al. (2024) reported that EVs derived from *Lactobacillus rhamnosus* GG potently stimulate the phagocytosis of macrophages, particularly in the simulated intestinal environment. Mechanistically, the FPR1/2 signaling pathway and its downstream pathways PI3K-Akt-MARCO and NADPH-ROS were significantly activated after treatment with EVs at pH 8. This activation leads to the upregulation of scavenger receptor MARCO (Macrophage receptor with collagenous structure) expression and increased production of reactive oxygen species against bacteria within macrophages (Jiang and Huang, 2024; Zhu et al., 2024). The intestinal microbiota interacts with the immune system through OMVs (Qiu-Sha et al., 2019). OMVs, such as those produced by *E. coli* ECOR12, modulate innate immunity via the nucleotide-binding oligomerization domain (NOD) related signaling pathway, priming the immune system to regulate inflammation and maintain intestinal equilibrium (Fábrega et al., 2017; Zyrek et al., 2007). These vesicles deliver bioactive molecules to cytosolic NOD receptors, which are key in detecting microbial patterns and shaping microbiome composition. NOD1, which is expressed in the intestinal epithelium, senses *E. coli* ECOR12-derived OMVs, triggering inflammasome assembly (e.g., NAIP3) and activating signaling cascades such as the nuclear factor kappa B (NF-κB) and Mitogen-activated protein kinase (MAPK) cascades. This process promotes cytokine production, orchestrating immune and inflammatory responses crucial for intestinal homeostasis (Cañas et al., 2016; Philpott et al., 2014). Probiotic modulation of NOD1 by OMVs highlights a sophisticated mechanism vital for microbiome balance and immune regulation (Fig. 1) (Cao, 2016; Nozaki et al., 2022).

## Proliferation and Differentiation of Regulatory T Cells and Suppression of Cytotoxic T Cells

**Fig. 2.**
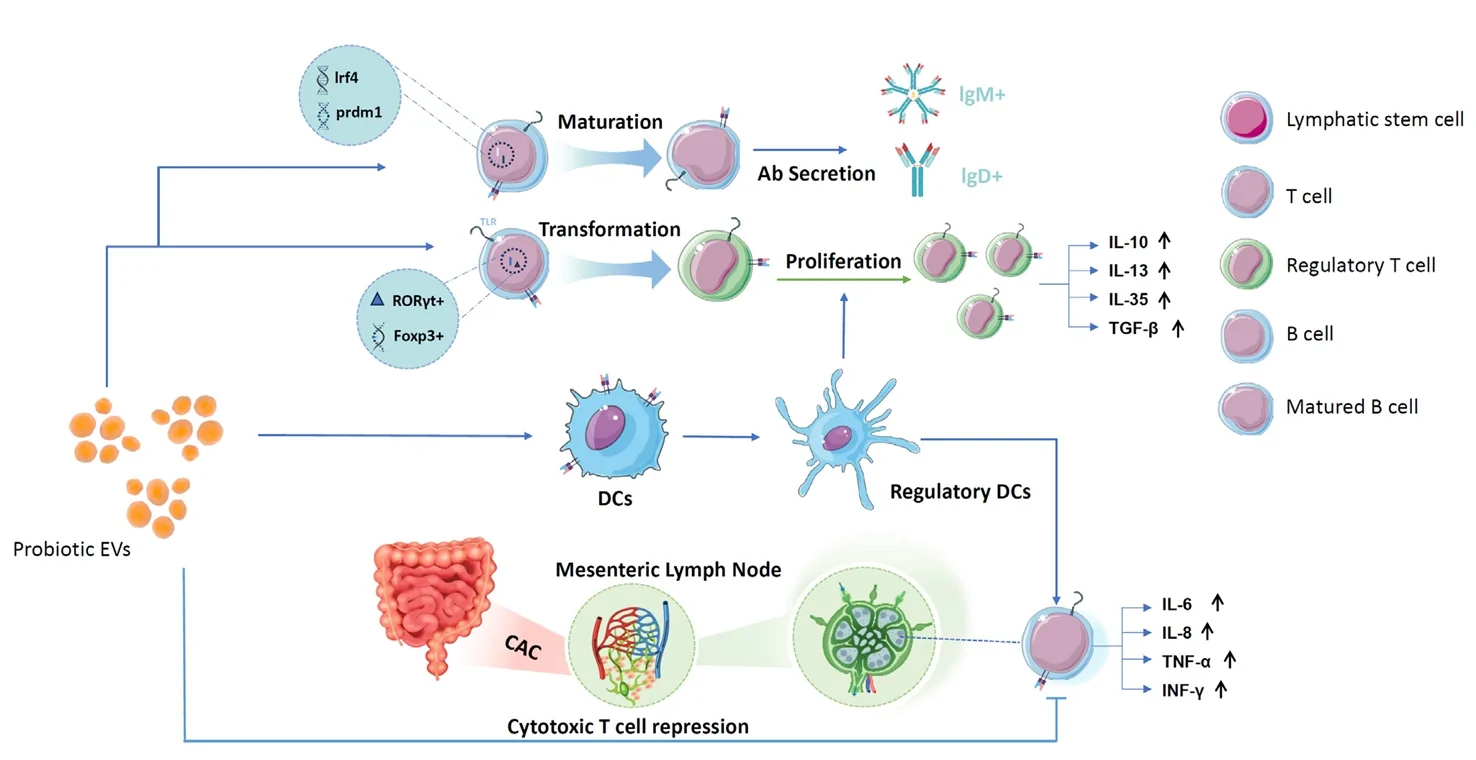
The role of probiotic-derived extracellular vesicles on adaptive immunity. Probiotic EVs interact with dendritic cells (DCs: promote DC maturation and transformation, and influence the activation, proliferation, and differentiation of T and B cells. They directly act on B cells to promote their maturation, proliferation, and secretion of specific antibodies; directly act on T cells to activate the transcriptional expression of *RORγ*^+^ and *Foxp3*^+^, and induce the differentiation of regulatory T cells. Additionally, they promote the conversion of DCs into regulatory DCs, thereby enhancing the proliferation of Tregs and the secretion of anti-inflammatory factors.

Probiotic-derived EVs enhance the differentiation and proliferation of regulatory T cells (Tregs) through various mechanisms involving fine-tuned genetic regulation. First, miRNAs contained in probiotic EVs, such as miR-142-3p and miR-150-5p, can reduce the expression of pro-inflammatory cytokines and may regulate immune responses and inflammation by targeting specific signaling molecules (Zhou et al., 2018). These miRNAs modulate the differentiation of Treg cells by affecting dendritic cells (DCs). Signaling pathways such as the mTOR and NF-κB pathways play crucial roles in regulating the balance between Tregs and CTLs, and probiotic EVs may activate genes related to regulatory Treg cells, such as *Foxp3* and *RORγt*, by influencing these pathways to achieve differentiation and balance (Gerondakis et al., 2014; Round and Mazmanian, 2010). *Bacteroides fragilis* releases Polysaccharide A (PSA) via OMVs, which mediate immune regulation and prevent experimental colitis. DCs detect OMV-associated PSA through Toll-like receptor 2 (TLR2), resulting in enhanced development of Tregs and the production of anti-inflammatory cytokines. This signaling process in DCs depends on the Growth Arrest and DNA-Damage-Inducible protein (Gadd45α). Notably, OMV-treated DCs provide protection against colitis, whereas Gadd45α-deficient DCs fail to induce Tregs or suppress inflammation. These findings demonstrate that OMV-mediated PSA delivery enhances immune tolerance and reveal a novel mechanism of communication between the microbiota and the mammalian immune system (Shen et al., 2012). The absence of *Bifidobacterium* is linked to systemic inflammation and immune dysregulation in early life. The *Bifidobacterium longum* subspecies *infantis* EVC001 has been shown to reduce intestinal inflammation by promoting the expression of human milk oligosaccharide-utilizing genes, effectively modulating gut Th2 and Th17 responses in breastfed infants (Henrick et al., 2021). Lp_MVs have been shown to possess the remarkable ability to fine-tune adaptive immune responses. In particular, the Lp25_MVs strain has been shown to significantly influence T-cell differentiation, driving the development of T helper cells toward the Th1 phenotype, which is essential for enhancing cell-mediated immunity against intracellular pathogens (Kurata et al., 2022). Probiotic-derived EVs alleviate inflammatory responses by reducing the infiltration of M1-type macrophages. Similarly, they can also relieve inflammation by reducing the infiltration of CTLs in tissues (Fig. 2).

## Immune Intervention with B Cells

Probiotic-derived EVs affect B cells by promoting antibody production and influencing B cell differentiation. Probiotic-derived EVs stimulate B lymphocytes, modulating signal transduction pathways such as the NF-κB and MAPK pathways, which affect B cell differentiation and the production of antibodies, particularly IgA, potentially related to the internalization of probiotic EVs in the gut (Thomas and Versalovic, 2010; van Zyl et al., 2020). Second, studies have indicated that probiotics can alter immune-related enzyme activities, promoting the differentiation of B lymphocytes into plasma cells and stimulating or enhancing the production of antibodies under pathological conditions (van Zyl et al., 2020). Additionally, probiotic EVs can interact with DCs, influencing T cell activity and cytokine production, thereby indirectly affecting B cell functions and enhancing anti-inflammatory and anti-infectious capabilities (Gerondakis et al., 2014; Qian et al., 2012). This regulatory effect may indirectly influence B cell differentiation and function. In summary, probiotic EVs impact B cells through direct and indirect mechanisms, including the promotion of antibody production and the influence on B cell differentiation into plasma cells, thereby playing a significant role in modulating immune responses and enhancing gut barrier functions. MVs of *Bacillus subtilis* (*B. subtilis*) have been shown to modulate the expression of critical genes such as Irf4 and the Prdm1 gene cluster, which are crucial for the maturation and antibody production of B lymphocytes (Perdiguero et al., 2019). Moreover, *B. subtilis* derived MVs (*B. subtilis*_MVs) have been found to increase the frequency of IgM-producing cells, suggesting their role in promoting B cell proliferation and differentiation. The modulation of IgM+ and IgD+ B cell subsets suggests that *B. subtilis*_MVs influence key processes, such as antibody affinity maturation and class-switch recombination, which are essential for generating precise, protective antibody responses to pathogens (Toyofuku et al., 2019). Notably, *B. subtilis*_MVs has unique effects on IgM-producing cells compared with those of other *Bacillus* species, such as *Bacillus megaterium*, highlighting its species-specific nature and potential as a targeted immunomodulator (Vicente-Gil et al., 2024).

*Lactobacillus paracasei* and *L. paracasei*-derived MVs (Lpc_MVs) have emerged as potent immunomodulators that orchestrate T cell differentiation toward Tregs and enhance B cell activation and antibody secretion. These MVs delicately balance the humoral immune response by modulating the expression of critical genes such as Irf4 and the Prdm1 gene cluster (Sandanusova et al., 2024). The modulation of these genes, in turn, influences signaling pathways, including the NF-κB, MAPK, Akt/PI3K, and STAT pathways, which are essential for B cell proliferation and differentiation (Guo et al., 2024). Lpc_MVs increase the frequency of IgM-producing cells, suggesting their role in promoting B cell proliferation and differentiation. Furthermore, the modulation of IgM+ and IgD+ B cell subsets by Lpc_MVs indicates their influence on key processes, such as antibody affinity maturation and class-switch recombination, which are crucial for generating precise, protective antibody responses to pathogens (Strzelec et al., 2023).

## Intervention of Probiotic EVs in Intestinal Barrier Protection

The innate intestinal barrier comprises the mucosal epithelium, lamina propria, and muscle layers, which protect against pathogens via tight junctions. Under normal conditions, these microorganisms coexist in a balanced, stable equilibrium, safeguarding the integrity of the intestinal ecosystem. This balance is crucial for intestinal health and contributes to the host’s metabolic and immune well-being. Moreover, by increasing the expression of tight junction proteins, EVs enhance intestinal barrier integrity, reduce intestinal permeability and the risk of metabolic endotoxemia, and prevent the translocation of pathogens and entry of harmful agents such as LPS into the bloodstream.

EVs produced by beneficial gut bacteria play a crucial role in maintaining the integrity of the intestinal barrier. These vesicles, which are laden with biologically active compounds such as short-chain fatty acids (SCFA) and proteins, interact with intestinal epithelial cells to strengthen the mucus layer. SCFAs, especially butyrate, promote intestinal cell turnover, mucosal repair, and epithelial barrier resilience. SCFAs are pivotal in maintaining gut health through diverse mechanisms. They enhance gut barrier function by upregulating tight junction proteins (e.g., occludin and claudin-1) and stimulating mucus production. Butyrate, a crucial SCFA, activates hypoxia-inducible factors to maintain the low-oxygen environment in the colon, supporting anaerobic symbionts and inhibiting pathogens. SCFAs modulate the immune response by promoting Treg differentiation via histone deacetylase inhibition or activation of G protein-coupled receptors, thereby reducing inflammation. Butyrate also polarizes macrophages toward an anti-inflammatory phenotype (M2) and enhances antimicrobial peptide secretion while decreasing the secretion of pro-inflammatory cytokines (e.g., IL-8 and IL-6) and increasing the secretion of anti-inflammatory factors (e.g., IL-10 and TGF-β1). Metabolically, butyrate serves as a primary energy source for colonic epithelial cells, supporting cell metabolism and differentiation through β-oxidation. It induces cell cycle arrest and apoptosis in colorectal cancer cells via histone deacetylase inhibition, a phenomenon known as the "butyrate paradox." Butyrate also promotes intestinal epithelial cell differentiation by inhibiting histone deacetylase or transcription Factor Sp1 activation, maintaining the structural integrity of the gut. SCFAs regulate gut motility and endocrine functions by stimulating enteroendocrine cells to secrete gut hormones such as GLP-1, PYY, and serotonin through GPR41/GPR43 activation or histone deacetylase suppression. Butyrate enhances electrolyte absorption and maintains gut water and salt balance through sodium-coupled transporters. In microbial interactions, SCFAs inhibit pathogen proliferation by lowering the gut pH and exerting direct antibacterial effects. They support beneficial bacteria by maintaining an anaerobic environment and providing metabolic products (e.g., ketone bodies). Butyrate increases histone acetylation, promoting the transcription of anti-inflammatory and barrier-related genes (e.g., TGF-β1 and MUC2) and influencing DNA methylation and protein phosphorylation (Ahmad et al., 2000; Hass et al., 1997; Martin-Gallausiaux et al., 2021). Although the detailed mechanism of intestinal protection is provided by SCFAs, EVs play an important "carrier role" in this process, which is the mainstream way in which probiotic EVs work.

**Fig. 3.**
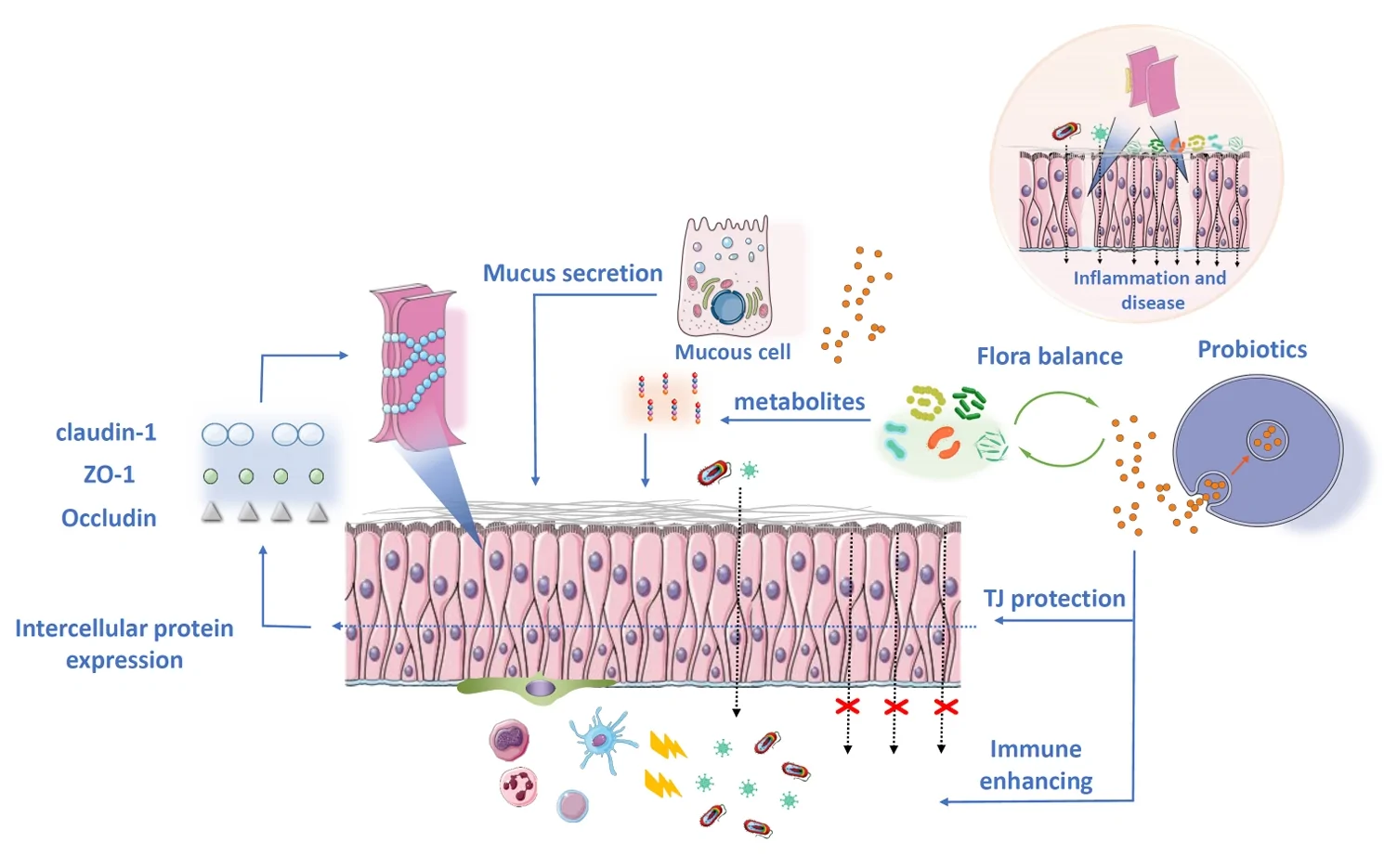
Intestinal barrier protection function and microflora balance contribution of probiotic-derived extracellular vesicles. Probiotic extracellular vesicles (EVs) strengthen the intestinal mucus layer and upregulate tight junction protein expression, thereby enhancing intestinal barrier function. They also modulate the host immune response through diverse immunomodulatory mechanisms, such as stimulating anti-inflammatory cytokine production and activating immune cells. Additionally, these vesicles influence the metabolism and stress response of intestinal microbes through signal exchange between bacteria and between bacteria and the host, promoting maintenance of intestinal flora balance.

Commensal bacteria-derived EVs exhibit immunomodulatory properties, enhancing intestinal barrier integrity by promoting the expression of immunomodulators such as IL-22. This stimulates mucus secretion from goblet cells, reinforcing the protective mucosal layer and supporting anti-inflammatory mechanisms (Hiippala et al., 2020). EcN_OMVs have been shown to strengthen the intestinal barrier by enhancing tight junction and modulating trefoil factor 3 (TFF3) expression, which further promotes ZO-1 translocation to junctions, restoring epithelial integrity and reducing inflammation-induced hyperpermeability (Fábrega et al., 2016, 2017). A separate study demonstrated that EcN_OMVs upregulated the expression of the tight junction protein ZO-1 in both healthy mice and dextran sulphate sodium-treated mice and further increased the expression of ZO-2 and claudin-14 in vitro (Behrouzi et al., 2020). Additionally, matrix metalloproteinase-9, an enzyme that exacerbates intestinal inflammation by disrupting tight junctions, is downregulated by EcN_OMVs (Fig. 3) (Fábrega et al., 2017). These findings highlight the regulatory role of probiotic OMVs in modulating bacterial interactions and strengthening the intestinal barrier.

## Probiotic EVs Regulate Flora Interactions

The functions of EVs can be broadly classified into two categories: those involved in bacteria–bacteria interactions and those related to bacteria–host communication. This dynamic equilibrium relies on a fine-tuned interplay of competition and cooperation among microorganisms, reflecting life’s evolutionary harmony. Each microbial participant, regardless of size, contributes critically to this stability. By facilitating signaling and mediating interactions, EVs ensure a functional and resilient microbiome, directly supporting gut integrity and host well-being. This delicate system underscores the profound interconnectedness of microbial life and human health.

EVs from diverse probiotic strains play a vital role in interbacterial communication and help their producer bacteria thrive within ecological niches. These vesicles act as a microbial defense mechanism by offering protection against a range of threats. For example, EVs can sequester bacteriophages, preventing them from interacting with bacterial cells and minimizing damage. They can also serve as decoys for antibiotics or contaminants that target bacterial membranes, effectively shielding bacteria from external harm. Probiotic *Bacteroides* species, for example, produce EVs containing beta-lactamases that confer antibiotic resistance, benefiting both the producer strain and neighboring bacteria (Stentz et al., 2014).

An intriguing study demonstrated that the effects of microbiota on EVs are reciprocal and bidirectional, challenging the notion that individual microbiota is influenced solely by EVs. Under simulated in vivo culture conditions, *Bifidobacterium longum* subsp. *longum* BG-L47 significantly promoted the growth and production of MVs from *Lactobacillus reuteri* DSM17938 (Ermann Lundberg et al., 2024). Additionally, the presence of BG-L47 increased the activity of 5'-nucleotidase in *Lactobacillus reuteri* derived MVs (Lr_MVs). In terms of immune modulation, Lr_MVs cocultured with BG-L47 exhibited a stronger antagonistic effect on the Transient Receptor Potential Vanilloid 1 (TRPV1) and increased the expression of immune development markers such as IL-6 and IL-1β in an in vitro model (Ermann Lundberg et al., 2024). These findings underscore the bidirectional and mutually complementary interaction between *Bifidobacterium longum* subsp. *longum* BG-L47 and Lr_MVs, offering valuable insights for the development of innovative probiotic formulations.

## Antitumor Capability of Probiotic EVs

Cancer is not only a major burden on the health of people in the United States and China (Maomao et al., 2022; Xia et al., 2022) but also the second leading cause of death in the U.S. and globally (Browne, 2023; Fitzmaurice et al., 2017). Current methods for the clinical management of tumors, such as radiotherapy (Gong et al., 2021), chemotherapy (Mitsuma and Ando, 2022), surgery (Takiguchi et al., 2020), and small-molecule targeted therapy (Wang et al., 2022), still face substantial challenges, including tissue toxicity, the development of drug resistance, and high recurrence rates (Hanahan and Weinberg, 2011; Karamitopoulou et al., 2023). Recent evidence suggests that probiotics may increase the efficacy of immunotherapies by reducing the side effects of cancer treatments and aiding tumor suppression (Liu et al., 2022; Thomsen and Vitetta, 2018). This raises the potential for integrating microbiological therapies with immunotherapies to improve cancer treatment outcomes (Mager et al., 2020). These findings underscore the burgeoning interest in the role of the microbiome in cancer management and the prospect of harnessing probiotics as a noninvasive strategy for cancer prevention.

EVs have already been utilized in tumor immunotherapy for three key reasons. First, they have strong immunogenicity. Second, they are unable to proliferate in vitro. Third, they have the ability to enhance antigen presentation (Furuyama and Sircili, 2021). Owing to their small size, EVs can easily pass through the gut and are readily internalized by DCs abundant in the intestinal epithelium, thereby activating the immune system and generating a broad repertoire of T cells (Tomasi et al., 2022). This internalization process is essential for initiating immune responses, as it triggers the activation of a diverse pool of T cells critical for immune surveillance and response.

Animal studies have also shown that enhancing intestinal barrier function with these probiotics can prevent the development of various cancers, including lung, breast, liver, and colorectal cancers (Bertocchi et al., 2021; Sun et al., 2023; Xue et al., 2018). Intriguingly, research by Ma et al. (2020) revealed that *Faecalibacterium prausnitzii* (*F. prausnitzii*) - derived OMVs (Fp_OMVs) can suppress the proliferation of breast cancer cells by targeting the IL-6/STAT3 signaling pathway. However, these findings are primarily based on preclinical data, and the lack of human data limits the direct extrapolation of these results to clinical settings. Additionally, the composition of OMVs may vary, which could influence their therapeutic effects. Therefore, further research is needed to fully elucidate the mechanisms and clinical relevance of Fp_OMVs in cancer treatment. Future research is urged to further elucidate the mechanisms underlying the therapeutic effects of Fp_OMVs and establish their clinical relevance. Specifically, studies involving human subjects are essential for validating these preclinical findings and addressing the potential variability in OMVs composition. In lung cancer studies, Battal et al. (2014) suggested that Fp_OMVs modulate the immune response by upregulating anti-inflammatory cytokines such as IL-10 and TGF-β2 while suppressing pro-inflammatory cytokines such as IL-6, TNF-α, and TNF-β. This modulation is mediated through the output of EVs. Moreover, the presence of a high density of *F. prausnitzii* in the gut microbiome of post-surgery patients with colorectal cancer is correlated with improved overall survival rates, a critical metric in cancer research (Kaźmierczak-Siedlecka et al., 2022). Amuc_1000 can play a key role in regulating the host immune response and maintaining intestinal barrier integrity, thus contributing to cancer prevention as well (Ottman et al., 2017). Xu et al. (2020) reported that *A. muciniphila* can influence glycerophospholipid metabolism, potentially affecting the expression of immune cytokines such as IFN-γ and IL-2 within the tumor microenvironment. This metabolic impact may enhance the efficacy of anti-PD-1 antibodies, a significant advancement in cancer immunotherapy. While the direct therapeutic effects of these bacteria on cancer have not yet been conclusively demonstrated, the diminished presence of both Fp_OMVs and *A. muciniphila* derived OMVs under cancerous conditions suggests their potential utility in cancer prevention and suppression (Kaźmierczak-Siedlecka et al., 2022; Ma et al., 2020).

EVs can elicit a strong IFN-γ response and activate T cell-mediated anti-tumor immunity. However, the same IFN-γ response can also induce the expression of programmed death ligand 1 (PD-L1) on tumor cells, potentially diminishing the effectiveness of immunotherapy by suppressing T cell activity (Kim et al., 2017; Rožman and Švajger, 2018). In a groundbreaking study, Li et al. (2020) utilized genetic engineering to create a ΔmsbB mutant *E. coli* strain known for its reduced endotoxin toxicity. This strain was used to generate plasmids that produce OMVs-PD1, which are engineered vesicles that display PD1 on their surface. These vesicles can bind to PD-L1, targeting the PD1/PD-L1 immune inhibitory axis. This interaction not only aids tumor internalization and reduction but also helps prevent T cell exhaustion. Furthermore, the binding of OMVs-PD1 to toll-like receptors can trigger the release of cytokines such as IL-6, IFN-γ, TNF-α, and IL-1β via feedback, enhancing the overall effect of anti-cancer immunotherapy. A similar immune response, utilizing the anti-PD-1 approach, has been observed with *Lactobacillus rhamnosus* GG in colorectal cancer, highlighting the broad potential of bioengineered probiotics in clinical cancer treatment (Lu et al., 2023). However, caution is important when interpreting these results, as the clinical application of such bioengineered probiotics requires further validation in human studies.

## More Biological Potential of Probiotic EVs

**Fig. 4.**
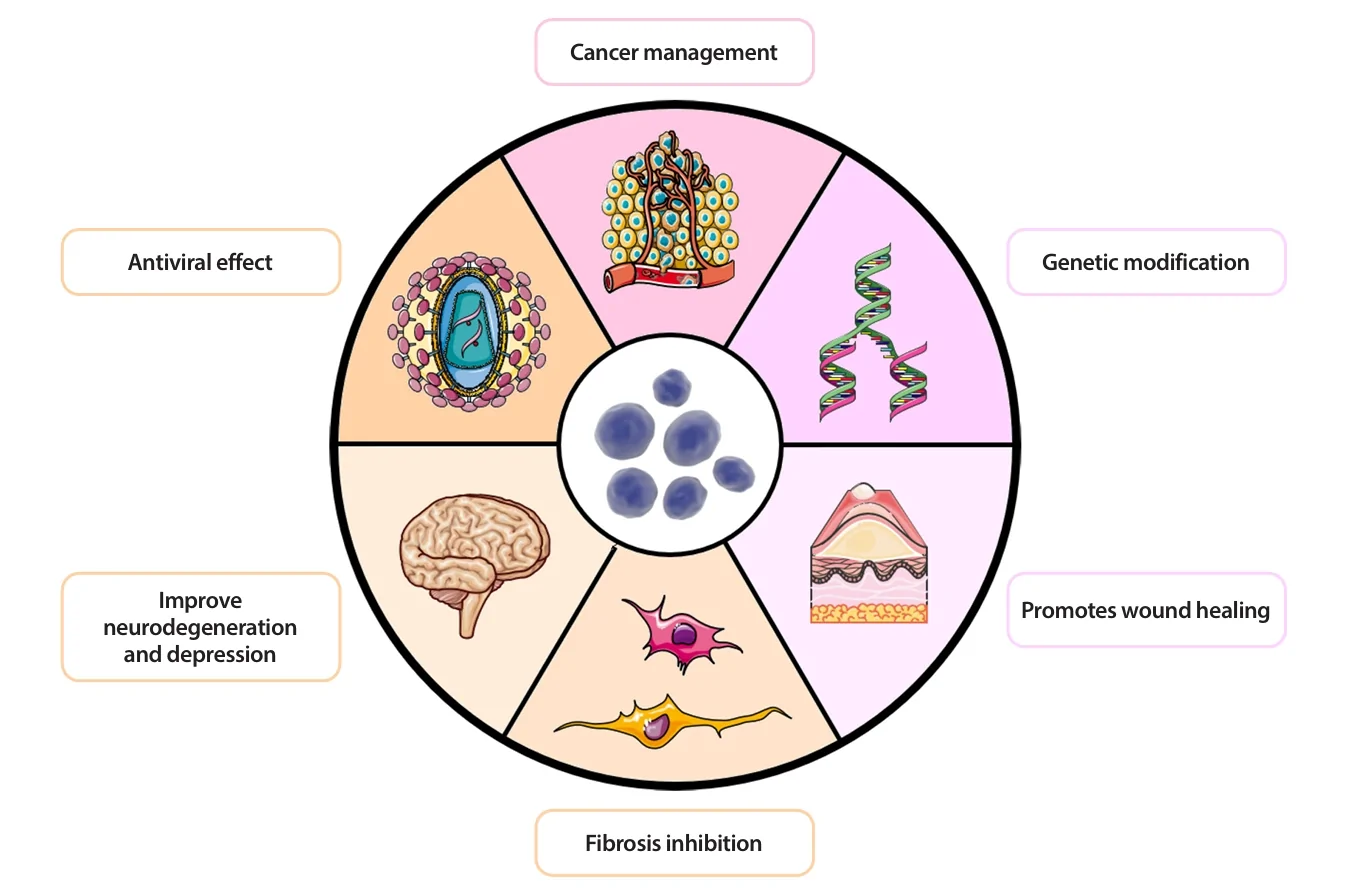
Pleiotropy of probiotic EVs and their mechanisms of action in disease treatment. Probiotic EVs exhibit antidepressant effects by modulating the gut-brain axis, suppressing pro-inflammatory cytokines, and influencing epigenetic factors. Additionally, these vesicles demonstrate the ability to combat HIV-1 infection by inhibiting viral replication and preventing the virus from binding to host cells. In the context of neurodegenerative diseases, EVs enhance cognitive function and ameliorate the pathology of Alzheimer's disease through the upregulation of neurotrophic and epigenetic factors. For fibrosis management, EVs promote wound healing and reduce scar formation by suppressing fibroblast proliferation and activating diverse signaling pathways. Furthermore, probiotic outer membrane vesicles exhibit anti-cancer properties by regulating immune processes.

Current clinical research has identified gut microbiota regulation as a promising therapeutic target for mental disorders, with associations between microbial composition alterations and symptom severity in depression, anxiety, and bipolar disorder (Fig. 4). Probiotic-derived EVs are thought to exert antidepressant-like effects through a plethora of intricate mechanisms. These EVs are believed to influence the “gut-brain axis,” a network connecting the neural, endocrine, and immune systems, and may therefore modulate mood, behavior, and cognition (Zhou and Foster, 2015). The gut microbiota involved in this axis are crucial for maintaining brain function, which plays a role in preventing and treating mental disorders, including depression and anxiety (Liang et al., 2018; Yuan et al., 2021). The anti-inflammatory properties of EVs are particularly notable in this context. These vesicles help mitigate neuroinflammation caused by chronic stress, primarily by dampening pro-inflammatory cytokines, thereby reducing depressive symptoms (Choi et al., 2022). Additionally, EVs carry epigenetic modulators such as DNA methyltransferases and histone deacetylases, enabling them to recalibrate gene expression related to depression without altering the underlying DNA sequence. This ability of EVs to influence gene expression highlights their potential as therapeutic agents for depression and other mood disorders (Choi et al., 2019, 2022). In a study by Choi et al. (2019), depression was induced in mice, and the administration of Lp_MVs led to an increase in brain-derived neurotrophic factor expression in hippocampal neurons, resulting in antidepressant-like effects. Similarly, OMVs derived from *A. muciniphila* play a key role in modulating the gut microbiota, which in turn influences neurotransmitter production and brain-derived neurotrophic factor levels, both of which are critical to the pathophysiology of depression (Ding et al., 2021). These EVs also demonstrate anti-inflammatory effects, reducing neuroinflammatory processes that contribute to depressive symptoms (Deng et al., 2015). Metabolomic studies have revealed that the OMVs of *A. muciniphila* can alter the levels of serum metabolites such as β-alanyl-3-methyl-l-histidine and edaravone—which are linked to synaptic function and mood regulation (Ding et al., 2021; Lee and Xiang, 2018). Furthermore, *A. muciniphila* derived OMVs may help rebalance the neuroendocrine system by modulating the “gut-brain axis” and mitigating the effects of stress on the hypothalamic-pituitary-adrenal axis (Spencer and Deak, 2017). These findings highlight the promising therapeutic potential of *A. muciniphila* derived OMVs in treating depression, offering a new avenue for the development of next-generation mental health therapies. Other probiotic-derived EVs, especially those from specific *Bifidobacterium* species, have also been linked to antidepressant effects, primarily due to the metabolites they carry (Jach et al., 2023). In a recent study by Kwon et al. (2023), Lpc_MVs were shown to counteract the alterations in neurotrophic factor expression caused by amyloid-beta (Aβ), a hallmark of Alzheimer’s disease. Lpc_MVs improved cognitive function and Aβ pathology in Tg-APP/PS1 model mice by increasing the expression of key epigenetic factors, such as methyl-CpG binding protein 2 and sirtuin 1. Additionally, Lpc_MVs increased the expression of Aβ-degrading enzymes, reducing Aβ accumulation in the brain. These findings suggest that probiotics and their derivatives may offer new therapeutic strategies for neurodegenerative diseases through epigenetic mechanisms.

*Lactobacillus druckerii* derived MVs (Ld_MVs) have demonstrated substantial efficacy in inhibiting hypertrophic scar fibrosis, with both in vitro and in vivo evidence supporting their therapeutic potential. In vitro studies revealed that Ld_MVs significantly reduce the expression of key fibrosis markers, including collagen I/III and α-smooth muscle actin (α-SMA), in fibroblasts isolated from hypertrophic scars while simultaneously inhibiting fibroblast proliferation (Han et al., 2023). In vivo experiments utilizing a scleroderma mouse model further revealed that Ld_MVs decrease α-SMA expression, leading to a reduction in scar formation (Zahmatkesh et al., 2022). Additionally, in an excisional wound healing mouse model, Ld_MVs promoted skin cell proliferation, enhanced neovascularization, and accelerated overall wound healing (Yu et al., 2024). Proteomic analysis suggested that Ld_MVs may inhibit fibrosis in hypertrophic scar by activating multiple signaling pathways, including the MAPK pathway, adrenergic signaling, NOD-like receptor signaling, and pathways related to Cushing's syndrome, salivary secretion, and bile secretion (Han et al., 2023; Housmans et al., 2022). The MAPK pathway, known for its role in regulating cellular growth and proliferation, is particularly important. Ld_MVs appear to modulate fibroblast proliferation—both in hypertrophic and normal skin fibroblasts—via this pathway. These findings indicate that Ld_MVs hold significant promise not only for treating hypertrophic scars but also for potential application in other fibrotic diseases, suggesting a novel approach for fibrosis management.

In conclusion, EVs represent a promising therapeutic frontier in medicine, with the potential to revolutionize various fields of biomedicine. While numerous challenges remain, such as optimizing EVs isolation and delivery methods and understanding their full range of mechanisms, the multifaceted benefits of EVs in disease management are undeniable. Emerging research, particularly on probiotic-derived EVs, reveals their potential in cancer treatment, viral infection control, disease prevention, and even neuromodulation. Despite the current nascent state of research, the evidence highlights the salutary role of these vesicles in promoting human health. Moving forward, a deeper understanding of the pathways and mechanisms by which EVs exert their effects will be critical in unlocking their full therapeutic potential. This knowledge will not only broaden our understanding of how probiotics interact with the body but also pave the way for more targeted and effective treatments across a wide range of diseases. As the field progresses, probiotic-derived EVs could become a cornerstone in personalized and precision medicine.

Studies have shown that probiotic EVs may exhibit antiviral effects. *Lactobacillus vaginalis* plays a crucial role in preventing HIV-1 transmission, with the inhibition of HIV-1 infection linked to several proteins and metabolites found in MVs. The protective effects of lactic acid bacteria against HIV-1 are partially mediated by their MVs. Certain strains of *Lactobacillus vaginalis*-derived MVs were shown to exert antiviral effects in vitro by inhibiting HIV-1 replication and impairing viral entry into and attachment to human CD4+ T cells (Ñahui Palomino et al., 2019).

## EVs Biogenesis and Production

The biogenesis of EVs is influenced by a variety of factors, including environmental conditions, nutrient availability, physical parameters, and the bacterial growth stage. These factors can impact on the contents, production rates, and purities of EVs. EVs can be released during normal bacterial growth or under stressful conditions, although the precise mechanism of their formation remains unclear. In natural wild colonies, EVs are produced at relatively low concentrations and quantities. However, under certain conditions, such as temperature shifts, external stressors, quorum sensing, or exposure to antibiotics, the production of EVs can be significantly increased. In vitro, engineered bacteria have been utilized to increase EVs production, offering a controlled approach to increase the yield and efficacy of these vesicles for therapeutic or research purposes.

Under normal growth conditions, bacteria release mainly EVs through blebbing, a process in which the outer membrane protrudes outward without the use of ATP (Jin et al., 2022). EVs can also be released in large amounts because of cell death or lysis. There are several models explaining blebbing. One model posits that when membrane biogenesis is faster than cell wall growth, disrupting or rearranging the covalent linkages between the membrane and the cell wall can lead to EVs formation and hypervesiculation in some bacterial species (Weyant et al., 2023). Weyant et al. (2023) induced a mutation in the *nlpl* gene of *E. coli*, which encodes an outer membrane lipoprotein for peptidoglycan crosslinking and produces many vesicles.

It should be noted that bacterial outer membrane proteins do not necessarily exist within the EVs they release. Although the composition of EVs is theoretically similar to that of bacterial outer membranes—encompassing outer membrane proteins, lipids, nucleic acids, and other components—not all outer membrane proteins are encapsulated into EVs. The biosynthesis and loading of EV components are a highly complex process, influenced by various factors such as bacterial type, growth conditions, and environmental factors. Furthermore, many aspects of the selective incorporation of outer membrane proteins into EVs, as well as the mechanisms governing this selection, remain poorly understood (Katsir and Bahar, 2017).

Moreover, certain extracellular signaling molecules that can enhance blebbing have been identified, but most related research has been conducted on pathogens instead of probiotics. For example, *Pseudomonas aeruginosa* synthesizes the Pseudomonas quinone signal (PQS), which can increase membrane curvature, whereas surfactant-like phenol-soluble modulins, such as *Staphylococcus aureus*, may induce G+ vesicle budding (Mosby et al., 2022). Few studies have focused on how signaling pathways induce probiotic blebbing. However, research in this area is crucial for understanding the balance between probiotic MVs generation and the host intestinal environment and for improving the efficiency of artificial production of probiotic vesicles.

**Fig. 5.**
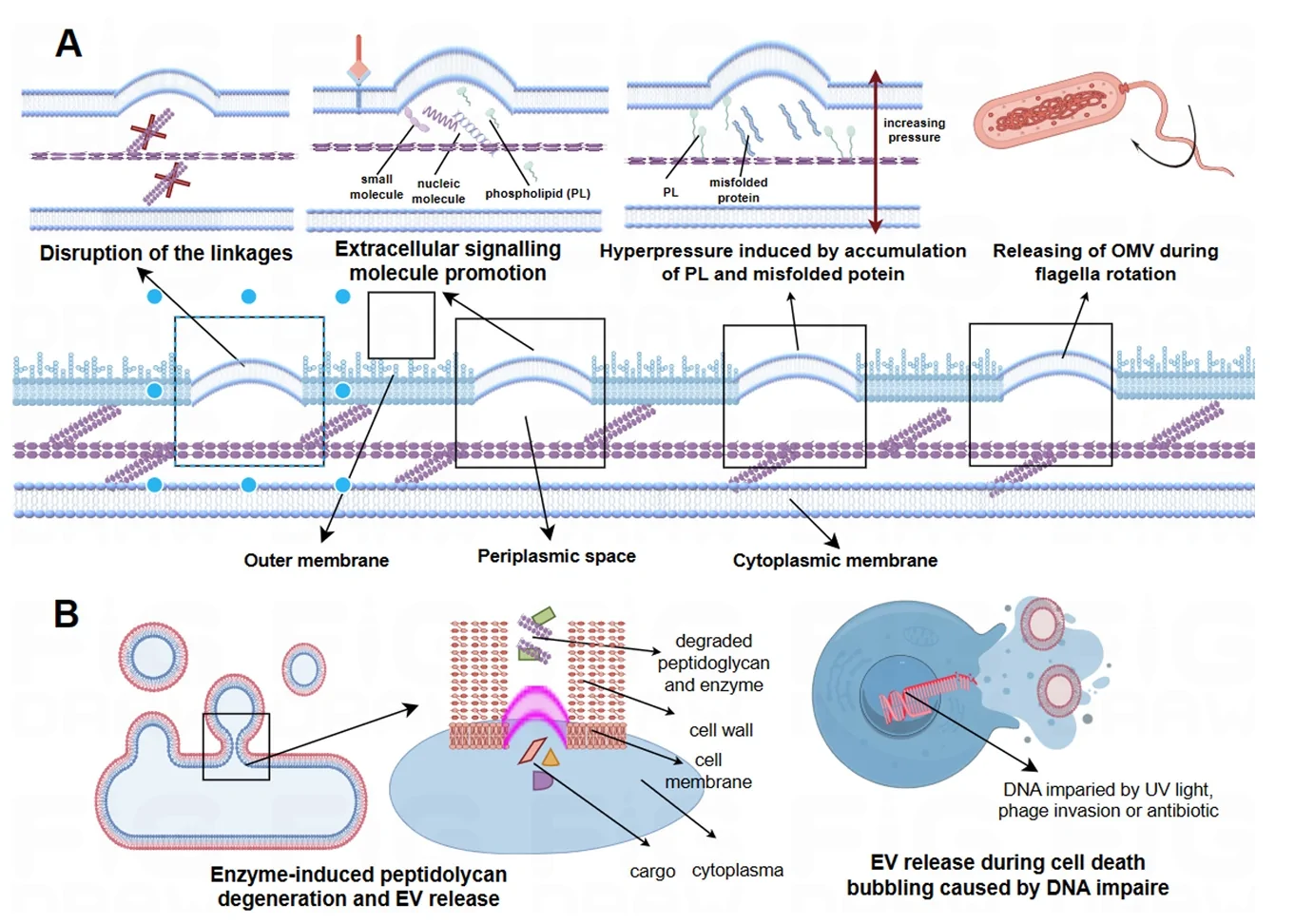
Patterns of extracellular vesicle biogenesis and release from Gram-negative and Gram-positive bacteria. (A) OMV production from Gram-negative bacteria. Disruption of the linkages between outer membrane and underlying cell wall and the extracellular molecule signalling would promote the OMV release from the cytoplasm. Meanwhile, accumulation of phospholipid and misfolded protein will increase the pressure between cytoplasmic membranes and outer membranes, which promote the transport of cargos and release of OMVs. In species with flagella, rotation would accelerate the release of OMVs. (B) EV production from Gram-positive bacteria. In cell death bubbling theory, degeneration of peptidoglycan by the endoenzyme and autoenzyme would promote the release of EVs from cytoplasmic membranes, while DNA damage caused by UV light, phage invasion and antibiotic administration would cause cell death and lysis which accelerate the generation of EVs.

In G- bacteria, the accumulation of peptidoglycan fragments or misfolded proteins can generate intracellular pressure, which exerts a stretching force on the outer membrane and results in hypervesiculation. Moreover, phospholipid (PL) accumulation in the outer leaflet of the outer membrane enhances the expansion and budding of OMVs. This accumulation of phospholipids can be regulated by the VacJ/Yrb ABC transport system, which transfers phospholipids from the outside of the OMVs to the inside (Batista et al., 2020). Mutants of the VacJ/Yrb ABC transport system could contribute to hypervesiculation and the production of PL-rich OMVs. Another mechanism is specifically found in bacteria with sheathed flagella. Membrane blebbing can be observed during flagella rotation, and one of the distinguishing features of these EVs is that they contain LPS, which can trigger bacteria‒host communication, such as immunomodulation or inflammation. These models highlight those substances, including LPS and peptidoglycan, play important roles in the biogenesis of EVs (Fig. 5A). Additionally, evidence has shown the relationship between genetic factors and EVs biogenesis, such as mutations (Kumar et al., 2022). However, explanations for EVs biogenesis at the gene level are still limited.

The biogenesis process of EVs in G+ bacteria remains unclear and is an area of active research. Researchers proposed that bubbling cell death (BCD) is the main mechanism for EVs biogenesis in G+ (Toyofuku et al. (2019)). In this theory, as G+ bacteria die, EVs are generated (Fig. 5B). The breakdown of peptidoglycan in the cytoplasmic membrane by enzymes enables and speeds up EVs release (Manning and Kuehn, 2013; Peregrino et al., 2024; Ton-That et al., 2004).

BCD has two methods. First, endolysins and autolysins can degrade peptidoglycan, causing explosive EVs release from weakened cell wall holes, as observed in *B. subtilis* treated with peptidoglycan-hydrolyzing enzymes or β-lactam antibiotics. The effectiveness of this bubbling depends on the cell wall thickness and degree of peptidoglycan hydrolysis. Second, DNA damage from antibiotic use, UV exposure, or prophage presence can lead to EVs production. For example, in *Lacticaseibacillus casei* and *Lactococcus lactis*, spontaneous prophage transversion via the holin–lysin system in standard culture results in the production of EVs, whereas the unstimulated control results in no EVs production. However, Sangiorgio et al. (2024) noted that while the BCD theory can explain cytoplasmic cargo loading into EVs and their release through the thick cell wall, it fails to convincingly explain the abundant lipid components in the EVs membrane, which differ from those of the parent bacteria. Recent genetic studies have explored the link between EVs release and genetic control. For example, increased expression of the *spf* and *spo0A* genes and the transcription factor σB can increase EVs release in *Streptococci* and *B. subtilis*, and increased *virR* gene expression promotes EVs generation in *Mycobacterium tuberculosis*. However, there is a lack of research on probiotics and their genetic patterns during EVs secretion (Brown et al., 2014; Resch et al., 2016; Sangiorgio et al., 2024).

## The Stage of the Field, Limitations and Development

Probiotic-derived EVs have drawn much attention for their role in the communication between bacteria and host cells in various systemic diseases. However, there are still significant research gaps.

The biogenesis of EVs is influenced by many factors. Although progress has been made, the exact production and release mechanisms remain unclear. While the regulated budding process from the membrane is commonly accepted, the associated molecular regulatory networks and environmental impacts need further study.

Probiotic EVs show great potential in therapy. They enable cross-kingdom communication and affect multiple physiological processes. Their immunomodulatory functions are crucial for conditions such as inflammatory bowel diseases and cancers. They also modulate the microbiota–gut–brain axis, offering new methods for psychiatric research and helping preserve intestinal barrier integrity. Additionally, they play a role in interbacterial communication in the gut microbiome, maintaining microbial homeostasis (Table 1).

For clinical application, the safety, side effects, and long-term efficacy of these EVs need further research. Considering the limitations of probiotics and the few adverse reactions associated with OMVs modification, additional modification technologies should be explored.

To reduce risks, EVs design and optimization must solve key issues. This includes minimizing the levels of immunogenic components such as LPS to reduce the risk of immune reactions. Precise dosage control is important to balance efficacy and safety, and maintaining batch-to-batch purity consistency is essential to avoid inconsistent therapeutic results.

Research on EVs is at the forefront of microbiology and medical science. Future research should focus on understanding their production, immune system interactions, and disease impacts. A multidisciplinary approach can improve EVs-based diagnostics and drug delivery. The development of computational models to predict the effects of EVs and design therapies, along with ensuring their safe and ethical clinical use, is crucial.

## Conclusion

Recent investigations into probiotic-derived EVs have revealed a rich reservoir of bioactive components. These molecular entities play pivotal roles in facilitating intricate cross-kingdom communication, thereby exerting a profound influence on a wide spectrum of physiological processes. This concise review focuses on the critical contributions of probiotic EVs to immune system modulation, with a particular emphasis on their indispensable role in preserving intestinal homeostasis and mitigating diverse pathological states.

The immunomodulatory functions of probiotic EVs transcend the mere activation of pattern recognition receptors such as TLRs. They intricately orchestrate the precise regulation of cytokine production and harmonize a balanced inflammatory response. By interacting with and modulating both host immune cells and the gut microbiota, these vesicles fortify the integrity and functionality of the intestinal barrier, safeguarding the overall health of the gastrointestinal tract.

The therapeutic implications of probiotic-derived EVs extend far beyond confinement by the gastrointestinal system. In oncological research, these compounds have emerged as promising areas of study owing to their remarkable anti-suitor properties. These attributes have the potential to increase the efficacy of immunotherapies while simultaneously reducing the adverse effects associated with traditional cancer treatments. Additionally, the modulation of the microbiota-gut-brain axis by EVs has highlighted their latent potential in the management of neuropsychological disorders, heralding new frontiers in psychiatric research.

As research in the domain of probiotic EVs continues to flourish, the prospects are exceedingly promising. A more in-depth comprehension of the underlying molecular mechanisms governing EVs-mediated effects is not only instrumental in deepening our understanding of their roles in health and disease but also serves as the bedrock for the development of innovative therapeutic strategies. The translational potential of these research findings underscores the burgeoning interest in this field and the unwavering commitment to harnessing the power of probiotics for the improvement of human health.

In summary, the therapeutic panorama shaped by probiotic EVs is expansive and multifaceted, presenting abundant opportunities to enhance our understanding and treatment modalities for a diverse array of diseases. As research progresses, EVs, which function as crucial messengers and modulators in the complex interplay between hosts and microbes, are destined to occupy a preeminent position in the vanguard of biomedical science.

## Figures and Tables

**Table 1.** Classification and potential functional characteristics of extracellular vesicles of probiotics

Bacterial species	Classification	Potential application
*A. muciniphila*	G-	Metabolism improvement in obese
		Intestinal barrier enhancement
		Anti-inflammation
		Immunoregulatory function
		Dysbiosis control
		Anti-tumor capability
		Developing drug delivery system
*O. splanchnicus*	G-	Anti-inflammation and immunoregulatory function
*EcN*	G-	Maintaining intestinal flora homeostasis
		Inflammatory bowel disease and ulcerative colitis therapy.
		New immunomodulator development
		Surface recombinant vaccine
		New generation of drug delivery system through vesicle
*B. fragilis*	G-	A natural biological therapy for intestinal inflammatory diseases
		Reinforcing immune homeostasis
*B. vulgatus*	G-	Immunomodulation and the preservation of a balanced gut microbiota
*F. prausnitzii*	G-	Anti-tumor effects or cancer therapy
		Immunoregulator with anti-inflammation agents
*L. vaginalis*	G+	Preventing HIV-1 transmission
*L. sake*	G+	Enhancement of mucosal immune against infection
*L. plantarum*	G+	Preventing scar formation
		Anti-tumor effects in colonic cancer
		Immunoregulator and anti-inflammation agents
		Neurological recovery in ischemic stroke patients.
*L. casei*	G+	Anti-infectious effects in intestine against pathogens
*L. rhamnosus*	G+	Immunomodulator and anti-inflammation
		Growth factor for injury treatment
		A potential adjuvant anti-tumor agent in immunotherapy
*C. butyricum*	G+	Immunomodulator and anti-inflammation
*B. bifidum*	G+	Immunomodulator and anti-inflammation

## Data Availability

Not applicable.
